# Supplementary figures and images for: Work-related psychosocial risk factors and psychiatric disorders: A cross-sectional study in the French working population
Source: PLoS One. 2020 May 26;15(5):e0233472. doi: 10.1371/journal.pone.0233472 (PMC7250420; doi:10.1371/journal.pone.0233472)

**Figure. Flow Chart of the online study about psychiatric disorder and PSRFs.**

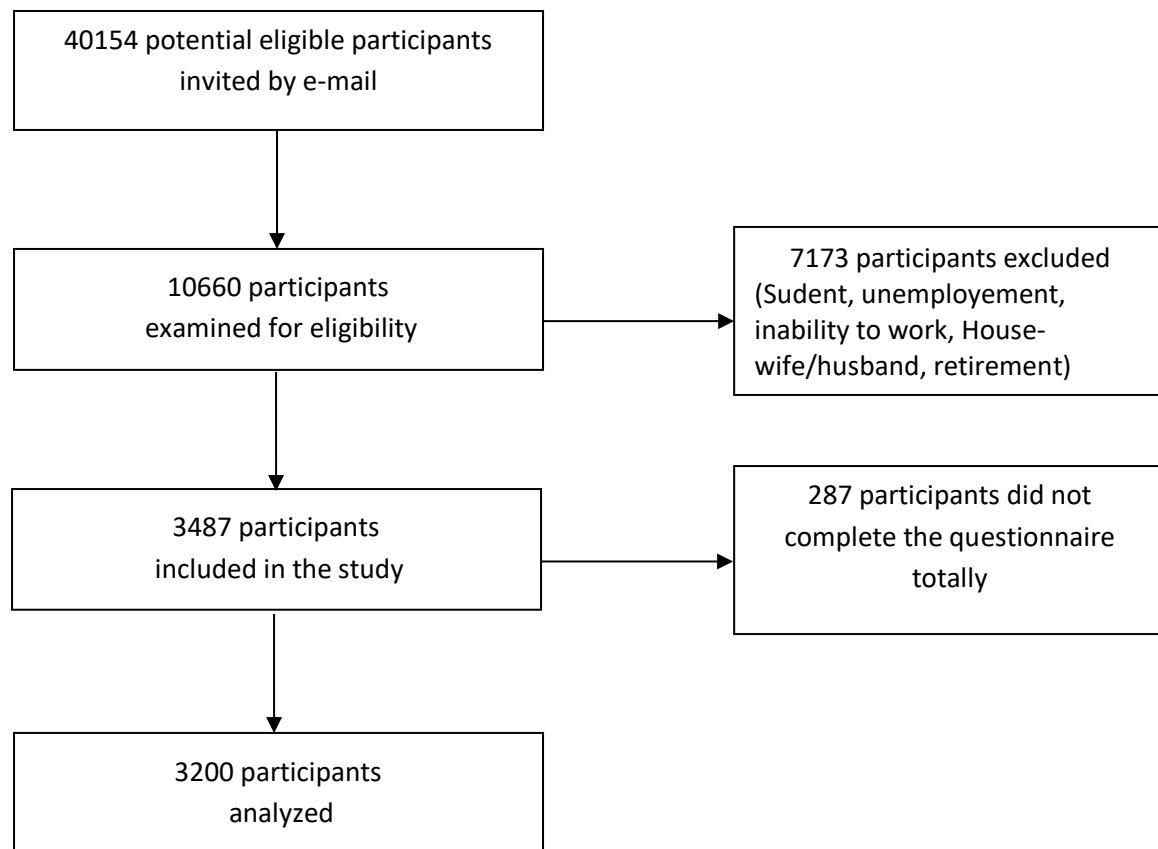

Supplement: S1 Fig — (PDF) [file pone.0233472.s001.pdf]
