# Supplementary material for: Work-related psychosocial risk factors and psychiatric disorders: A cross-sectional study in the French working population
Source: PLoS One. 2020 May 26;15(5):e0233472. doi: 10.1371/journal.pone.0233472 (PMC7250420; doi:10.1371/journal.pone.0233472)
Supplement: S1 Table — (PDF) [file pone.0233472.s002.pdf]

**Table. Psychosocial risk factors (PSRFs) assessed in the study.**

| | |
|---|---|
| **PSRF. 1** | My job consists of monotonous and repetitive tasks. |
| **PSRF. 2** | In my job. I must respect rigid procedures and I am closely supervised. |
| **PSRF. 3** | Company performance appraisals and promotions are fairly done. + (R) |
| **PSRF. 4** | I feel like I can't participate in my company's decision making process |
| **PSRF. 5** | The communication and information exchange process within my company is satisfactory.+ (R) |
| **PSRF. 6** | My work environment is pleasant. (inverse) |
| **PSRF. 7** | I don't exactly know what my corporate function is what is expected from me.+ |
| **PSRF. 8** | I handle an enormous amount of complex informations. |
| **PSRF. 9** | I have no problems handling my professional and private responsibilities. (R) |
| **PSRF. 10** | I am unable to plan what my job will be in 2 years |
| **PSRF. 11** | I need more time to do my job |
| **PSRF. 12** | I often have to deal with rude and/or aggressive people |
| **PSRF. 13** | In my job. I feel valued and recognized. (R) |
| **PSRF. 14** | When doing certain tasks in my job I often feel like I don't have enough training |
| **PSRF. 15** | My work atmosphere is unpleasant |
| **PSRF. 16** | My hierarchy not support and help me when needed.+ (R) |
| **PSRF. 17** | I spend a lot of time commuting for my job |
| **PSRF. 18** | I know that I can depend on the people I work with. (R) |
| **PSRF. 19** | In my job, making a mistake could have serious consequences. |
| **PSRF. 20** | My job does not make me feel useful nor gives me self esteem. |
| **PSRF. 21** | I have the necessary resources and technical means to correctly do my job. (R) |
| **PSRF. 22** | I have a good idea of my career prospects within the company.+ (R) |
| **PSRF. 23** | My job requires long periods of intense concentration. |
| **PSRF. 24** | My objectives are difficult to reach.+ |
| **PSRF. 25** | My work schedule is not flexible.+ |
| **PSRF. 26** | My work relationships are a source of satisfaction. (R) |
| **PSRF. 27** | The job I do requires that I constantly adapt to new things |
| **PSRF. 28** | I work in a noisy and hectic environment |
| **PSRF. 29** | Someone or some people at my workplace enjoy making me suffer. |
| **PSRF. 30** | I'm in charge of my work schedule and my breaks. (R) |
| **PSRF. 31** | I do not identify with my employer's corporate values.+ |
| **PSRF. 32** | I get along well with my hierarchy.+ (R) |
| **PSRF. 33** | I feel morally supported in my job. (R) |
| **PSRF. 34** | I am often interrupted during my work |
| **PSRF. 35** | My job often puts me in contact with clients/users |
| **PSRF. 36** | My job puts me into trying emotional situations |
| **PSRF. 37** | I'm proud of what I do in my job. (R) |
| **PSRF. 38** | Sometimes I feel afraid when I do my job |
| **PSRF. 39** | I am proud of my company.+ (R) |
| **PSRF. 40** | I feel like I often have to rush my work due to external constraints |
| **PSRF. 41** | In my job I am faced with constant change |
| **PSRF. 42** | I have a lot of leeway in my job. (R) |
| **PSRF. 43** | I am fearful for my professional future |
| **PSRF. 44** | I am satisfied with the compensation I receive for my job. (R) |
