# Supplementary material for: Work-related psychosocial risk factors and psychiatric disorders: A cross-sectional study in the French working population
Source: PLoS One. 2020 May 26;15(5):e0233472. doi: 10.1371/journal.pone.0233472 (PMC7250420; doi:10.1371/journal.pone.0233472)
Supplement: S2 Table — (PDF) [file pone.0233472.s003.pdf]

Table. Raw database, weighting factors and weighted database.

	Raw Data<br><br>N(%)	Weigth<br><br>N(%)	Weighted Data*<br>N(%)
**Total**	3200 (100)	1.000	3200 (100)
**Sex**			
Men	1796 (56.1)	0.923	1658 (51.8)
Women	1404 (43.9)	1.099	1542 (48.2)
**Age**			
18-24	96 (3.0)	2.733	262 (8.2)
25-34	695 (21.7)	1.059	736 (23)
35-44	940 (29.4)	0.916	861 (26.9)
45-59	1326 (41.4)	0.907	1203 (37.6)
>60	143 (4.5)	0.962	138 (4.3)
**PCS**			
Farmer	39 (1.2)	1.393	54 (1.7)
Independent	214 (6.7)	0.971	208 (6.5)
Executive manager	607 (19.0)	0.922	559 (17.5)
Intermediate Profession	866 (27.1)	0.956	828 (25.9)
Employees	914 (28.6)	0.972	889 (27.8)
Workers	560 (17.5)	1.182	662 (20.7)
**Region**			
Parisian Area	665 (20.8)	0.997	663 (20.7)
Parisian Area West	300 (9.4)	0.972	291 (9.1
Parisian Area East	237 (7.4)	1.000	237 (7.4)
North	195 (6.1)	0.953	186 (5.8)
West	455 (14.2)	0.950	432 (13.5)
East	285 (8.9)	0.955	272 (8.5)
South West	326 (10.2)	1.071	349 (10.9)
South East	405 (12.7)	0.989	400 (12.5)
Mediterranean	332 (10.4)	1.110	368 (11.5)
**Type of agglomeration**			
Rural	772 (24.1)	0.990	764 (23.9)
< 20 000 inhabitants	616 (19.3)	0.989	609 (19.0)
20 000 to 100 000 inh.	405 (12.7)	1.003	406 (12.7)
>100 000 inhabitants	901 (28.2)	1.007	907 (28.3)
Parisian Area	506 (15.8)	1.014	513 (16.0)

*Corresponding to the general population structure estimated by the INSEE 2013 study (for the demographic data) and from the Enquête Emploi 2012 study (for occupational data), both governmental studies.
