# Supplementary material for: Work-related psychosocial risk factors and psychiatric disorders: A cross-sectional study in the French working population
Source: PLoS One. 2020 May 26;15(5):e0233472. doi: 10.1371/journal.pone.0233472 (PMC7250420; doi:10.1371/journal.pone.0233472)
Supplement: S3 Table — (PDF) [file pone.0233472.s004.pdf]

Table: Results of the multiple logistic regression estimating the weight of the covariates on the GHQ-28 score for employees.

Variables	OR	95% CI		P value
Having a chronic disease	2.45	1.96	3.05	<0.001
Being a woman	1.57	1.27	1.94	<0.001
Age 45 to 59 vs 18 to 24 years	0.55	0.38	0.80	0.002
Working > 50 hours a week	1.71	1.16	2.50	0.007
Age > 60 vs 18 to 24 years	0.38	0.17	0.81	0.015
Staggered hours	1.22	0.95	1.55	0.114
Age 35 to 44 vs 18 to 24 years	0.76	0.53	1.10	0.143
Commuting time > 1 hr	0.85	0.68	1.06	0.143
Age 25 to 34 vs 18 to 24 years	0.78	0.54	1.13	0.190
Previous experience with unemployment	0.94	0.76	1.15	0.536
Working on the weekend	0.96	0.76	1.21	0.713
Working at night	1.01	0.77	1.31	0.959

OR = odds ratio; 95% CI =95% confidence interval
