# Supplementary material for: Work-related psychosocial risk factors and psychiatric disorders: A cross-sectional study in the French working population
Source: PLoS One. 2020 May 26;15(5):e0233472. doi: 10.1371/journal.pone.0233472 (PMC7250420; doi:10.1371/journal.pone.0233472)
Supplement: S4 Table — (PDF) [file pone.0233472.s005.pdf]

Table. Interactions between the 11 PSRFs and between sex and the 11 PRSFs.

Interactions	P-value	P value (Holm-Bonferroni)
"My job consists of monotonous and repetitive tasks." × "The communication and information exchange process within my company is not satisfactory "	0.008	0.553
"My job consists of monotonous and repetitive tasks." × "Sometimes I feel afraid when I do my job."	0.009	0.573
"I know that I can't depend on the people I work with." × "My job requires long periods of intense concentration."	0.009	0.573
"I have problems handling my professional and private responsibilities." × "My job requires long periods of intense concentration."	0.022	1
"Sometimes I feel afraid when I do my job." × "I am not satisfied with the compensation I receive for my job."	0.022	1
"Sometimes I feel afraid when I do my job." × "The communication and information exchange process within my company is not satisfactory".	0.025	1
"I know that I can't depend on the people I work with." × "The communication and information exchange process within my company is not satisfactory "	0.027	1
"I have problems handling my professional and private responsibilities." × "My job puts me into trying emotional situations"	0.037	1
"I have problems handling my professional and private responsibilities." × "My job does not make me feel useful nor gives me self esteem."	0.049	1
