# Supplementary material for: Work-related psychosocial risk factors and psychiatric disorders: A cross-sectional study in the French working population
Source: PLoS One. 2020 May 26;15(5):e0233472. doi: 10.1371/journal.pone.0233472 (PMC7250420; doi:10.1371/journal.pone.0233472)
Supplement: Attachment — Submitted filename: Response to reviewers.docx [file pone.0233472.s007.docx]

**Responses to reviewers**

Chevance et al. **Work-related psychosocial risk factors and psychiatric disorder: A cross-sectional study in the French working population.**

We thank all reviewers for their careful reading. Their comments greatly helped us improve our work. Please find in blue our comments and in green the modification of the manuscript.

**Reviewer #1:**

**This is a very interesting manuscript, very well written, and bringing original evidence.
This is indeed the first epidemiological French study made on a representative sample of the working population. Representativeness was achieved by using quota sampling. The methodology is very sound and follows international standards. The authors conducted a STROBE-compliant cross-sectional study of a representative sample of the French workers, allowing to estimate the prevalence of probable psychiatric disorders in this population and among workers exposed to work-related psychosocial risk factors.**

We thank Reviewer 1 for the positive comments and encouragements.

**I have few comments:**

**- It is surprising to include individuals aged 18 to 80 years, in a study on active workers in a country where the age of retirement is around 60 to 62 years old. Moreover, the study excluded retired people. Nearly 5% of the study sample is aged more than 60. Can you clarify this point please ?**

The eligibility criteria were:

- Individuals aged 18 to 80 years AND
- Having a job (e.g. any kind of job, including part-time/self-employment)

Exclusion criteria:

- Students
- Unemployed individuals
- Housewives/husband
- Retired people

A report of the French National Institute for Statistical and Economical Studies from 2018, showed that 5% of people aged [65-74] have an occupation (mainly men, with a good health condition, high education and living in the Parisian area). Top managers but also self-employed and farmers are overrepresented. Four profiles are described: employees with low education with part-time job, people with high education living in big cities, shopkeepers and farmers. (<https://www.insee.fr/fr/statistiques/3646000?sommaire=3646226>).

To collect data on the senior workers, we included people over the legal retirement age, who declared an occupation. They represented 4.5% of our whole sample (see supplementary table 2 page 3) which is consistent with the national statistics.

**- In this study, 42.6% of participants had a work duration of more than 39h a week, while the legal work duration is 35h a week. Who's is concerned by this ? Workers, independent, managers ?**

We performed supplementary statistics on this population of workers who reported working more than 39h per week in the table hereunder. Our results are consistent with the report of the French National Institute for Statistical and Economical Studies from 2017 with a mean work duration of 50.5 hours a week for self-employed and 39.1 hours a week for employees. (Please see figure 5 in https://www.insee.fr/fr/statistiques/3676634?sommaire=3696937)

|  | **Work duration ≥ 39 hours / week (n=1423)** | **Work duration < 39 hours / week (n=1777)** |
| --- | --- | --- |
| **Sex** |  |  |
| Male | 62.9 | 42.9 |
| Female | 37.1 | 57.1 |
| **Age, years** | 42.0 (10.7) | 40.9 (11.4) |
| **Number of children** |  |  |
| None | 50.1 | 54.5 |
| 1 | 23.4 | 20.5 |
| 2 | 21.2 | 18.9 |
| 3 | 4.6 | 4.9 |
| > 4 | 0.6 | 1.2 |
| **Highest educational degree** |  |  |
| < baccalaureate degree | 18.2 | 23.6 |
| Baccalaureate degree | 19.2 | 23.5 |
| Baccalaureate degree +2 years | 24.6 | 23.9 |
| Baccalaureate degree ≥ 3 years | 38.0 | 28.9 |
| **Annual household income (euros)** |  |  |
| < 15 000 | 7.8 | 13.7 |
| 15–24 000 | 17.4 | 22.8 |
| 24–36 000 | 25.1 | 27.0 |
| > 36 000 | 37.7 | 23.0 |
| No information | 11.9 | 13.5 |
| **Occupational status** |  |  |
| Independent worker | 16.2 | 7.4 |
| Employees | 83.8 | 92.6 |
| **Size of company (no. of employees)** |  |  |
| < 10 | 21.6 | 22.3 |
| 10–49 | 20.0 | 24.5 |
| 50–99 | 9.7 | 11.2 |
| 100–499 | 19.3 | 21.3 |
| > 499 | 17.1 | 16.0 |
| No information | 12.3 | 4.7 |
| **Activity sector** |  |  |
| Industry | 11.1 | 14.0 |
| Building | 15.2 | 8.3 |
| Trading | 12.1 | 11.7 |
| Transport | 5.5 | 5.6 |
| Insurance and real estate | 3.6 | 3.2 |
| Education, health and social work | 19.5 | 26.4 |
| Other services | 33.1 | 30.9 |
| **Duration of job** |  |  |
| < 6 months | 3.7 | 7.0 |
| 6 months–5 years | 33.2 | 33.9 |
| 6–10 years | 21.6 | 21.8 |
| > 10 years | 41.5 | 37.3 |
| **Commuting duration** |  |  |
| < 30 min | 55.6 | 61.6 |
| 30 min–1hr | 21.4 | 22.2 |
| > 1hr | 23.0 | 16.2 |

**- One major result is that the imbalance of work and personal life embodied the most strongest association with psychiatric disorder. This is a clearly very relevant result even from clinical point of view, as it is frequently observed in workers suffering from burnout.**

We agree with the reviewer that the imbalance of work and personal life is a major result of the study that echoed clinical practice. In this study we choose to focus on mental disorders and not on burn-out which is not part of the current mental disorder classification (neither the DSM-5 nor the ICM-10), as mentioned in the discussion.

**********************

**Reviewer #2:**

**This is a very interesting study about the estimation of the prevalence of probable psychiatric disorders in the working population, and the assessment of the proportion of people presenting a probable psychiatric disorder among people exposed to work-related psychosocial risk factors. A strength of the study is that it is conducted in a large sample representative of the French population. Only few points should be clarified.**

We thank the reviewer for acknowledging our work with positive comments.

**First, authors should define patients exposed vs unexposed regarding the psychosocial risk factors (PSRF). I think that a patient who is exposed to one PSRF given is a patient with a score of 1 regarding this PSRF in the list of 44 questions. But, authors should write it clearly.**

We add a sentence in the method section (paragraph 2) Measurement of psychosocial risk factors PSRF):

“Therefore, participants with a score of 1 to a given PSRF are called “participants exposed” to this PSRF in the results section.”

**Second, I do not understand well this sentence: “These two PSRFs were the most reported, with 75.7% and 70.5% of the sample reporting having a job that required constantly adapting to new things and 70.5% reporting often being in contact with customers/clients/users.” This sentence should be re written.**

We corrected the sentence as followed (results section, “exposure to work-related PSRFs):

“These two PSRFs were the most reported, with 75.7% of the sample reporting having a job that required constantly adapting to new things and 70.5% reporting often being in contact with customers/clients/users. “

**Except these two points, this manuscript is very clear with a consistent statistical analysis.
Moreover, results are very interesting.**
